# Detection of the Vibration Signal from Human Vocal Folds Using a 94-GHz Millimeter-Wave Radar

**DOI:** 10.3390/s17030543

**Published:** 2017-03-08

**Authors:** Fuming Chen, Sheng Li, Yang Zhang, Jianqi Wang

**Affiliations:** 1 Department of Biomedical Engineering, Fourth Military Medical University, Xi’an 710032, China; fumingfmmu@outlook.com; 2 College of Control Engineering, Xijing University, Xi’an 710123, China; sheng@mail.xjtu.edu.cn; 3 Center for Disease Control and Prevention of Guangzhou Military Region, Guangzhou 510507, China; zyfmmu@126.com

**Keywords:** radar measurement, vocal folds, auto-correlation function, voice activity detection, coherence analysis, vibration signal detection

## Abstract

The detection of the vibration signal from human vocal folds provides essential information for studying human phonation and diagnosing voice disorders. Doppler radar technology has enabled the noncontact measurement of the human-vocal-fold vibration. However, existing systems must be placed in close proximity to the human throat and detailed information may be lost because of the low operating frequency. In this paper, a long-distance detection method, involving the use of a 94-GHz millimeter-wave radar sensor, is proposed for detecting the vibration signals from human vocal folds. An algorithm that combines empirical mode decomposition (EMD) and the auto-correlation function (ACF) method is proposed for detecting the signal. First, the EMD method is employed to suppress the noise of the radar-detected signal. Further, the ratio of the energy and entropy is used to detect voice activity in the radar-detected signal, following which, a short-time ACF is employed to extract the vibration signal of the human vocal folds from the processed signal. For validating the method and assessing the performance of the radar system, a vibration measurement sensor and microphone system are additionally employed for comparison. The experimental results obtained from the spectrograms, the vibration frequency of the vocal folds, and coherence analysis demonstrate that the proposed method can effectively detect the vibration of human vocal folds from a long detection distance.

## 1. Introduction

Research on the vibration of vocal folds is important for evaluating speech production and other associated speech signal processing areas, particularly, human phonation and voice disorders. Vocal fold vibration is a highly complicated, compact three-dimensional vibration. The observation and measurement of vocal fold vibrations using equipment such as electroglottographs (EGGs) [1,2,3], video laryngoscopes [4], and high-speed video devices [5,6,7] have been successfully applied for studying the motion of the vocal cord tissues. However, these methods cannot directly express the vocal cord motion characteristics and they must be applied to the throat, causing discomfort to patients. Certain external monitoring devices such as microphones have also been employed for acquiring acoustic signals [8], however, the recorded acoustic signals are easily disturbed by the surrounding background noise, which can degrade the signal quality considerably.

Meanwhile, the electromagnetic (EM) radar sensor has been a promising alternative for various applications associated with phonation. In 1996, the EM radar sensor was first employed for speech coding, recognition, and synthesis [9]. In 1998, Holzrichter’s group developed a low-power EM radar sensor for speech-articulator measurements [10]. Subsequently, the EM radar was employed to measure tissue motions in the human vocal tract during voiced speech [11]. Thus, it was named the glottal electromagnetic micropower sensor (GEMS). In 2000, Titze et al. proved that GEMS signals are fundamentally similar to EGG signals. Moreover, it was shown that the wall movements of the vocal tract can be easily measured, enabling potentially useful applications for speech signals [12]. To further identify the vibration source of an articulator based on the EM radar sensor, Holzrichter et al. considered three sources of EM waves reflected from the laryngeal region. Their experimental results showed that the dominant source for reflected EM sensor signals are the vocal folds and that EM sensors effectively measure vocal fold movements [13]. In 2010, Chang’s group presented a continuous-wave radar system for extracting vocal vibration signals from human subjects [14]. Their results demonstrated that continuous-wave radar signals have excellent consistency in acoustic signals.

The above studies verified the effectiveness of the radar sensor in the measurement of the vibration signal from human vocal folds and of the glottal structure dynamics. However, in these studies, the radar sensor must be close to the human larynx. Further, the detection sensitivity is low, causing certain detailed vocal vibration signal information to be lost. In addition, it restricts the patient’s activity and causes discomfort and tension. Consequently, these shortcomings limit the development of methods for the noncontact detection of human vocal vibration signals.

Another area of consideration is the millimeter-wave radar sensor technology, which has been used for the detection of heart rate and respiration patterns over long distances [15,16]. A method of this type was employed to detect speech signals in our previous works [17,18,19]. These studies explored the principles and advantages of a 94-GHz radar for measuring human vocal vibration signals. Nevertheless, they only verified the effectiveness of the millimeter-wave radar in detecting speech signals, while focusing on enhancing the radar speech quality. In the present study, a 94-GHz millimeter-wave radar is employed to measure the vibration signal from human vocal folds. The high operating frequency of 94 GHz can be used to detect 5-µm displacements at a distance of 15 m and 20-µm displacements at 50 m [20]. In addition, the 94-GHz frequency can penetrate skin to a depth of 0.37 mm and human dermal thickness is between 2–3 mm; thus, the electromagnetic wave power is predominantly reflected off the skin. Nonetheless, the beam can penetrate optically opaque materials such as clothing, wood, and glass [21]. Therefore, the proposed radar sensor with a 94-GHz operating frequency is attractive for measuring vibration information from the skin over the larynx. To detect vibration signals from human vocal folds, an algorithm combining empirical mode decomposition (EMD) and the auto-correlation function (ACF) method is proposed. First, EMD is employed to suppress the noise of the radar-detected signal. Further, the ratio of the energy and entropy is used to detect voice activity in the radar-detected signal, following which a short-time ACF is employed to extract the human-vocal-fold vibration signal from the processed signal. For validating the method and assessing the performance of the radar system, a vibration measurement sensor and a microphone system are additionally employed for comparison. The experimental results obtained from the spectrograms, the vibration frequency of the vocal folds, and coherence analysis show that the proposed method can effectively detect the vibration of human vocal folds from a long detection distance.

The remainder of this paper is organized as follows: the 94-GHz millimeter-wave radar detection system for the vibration signal from the vocal folds is presented in Section 2, Section 3 and Section 4. The EMD-ACF algorithm is described in Section 5. The experimental results are presented and analyzed in Section 6.

## 2. Radar Detection System

**Figure 1:**
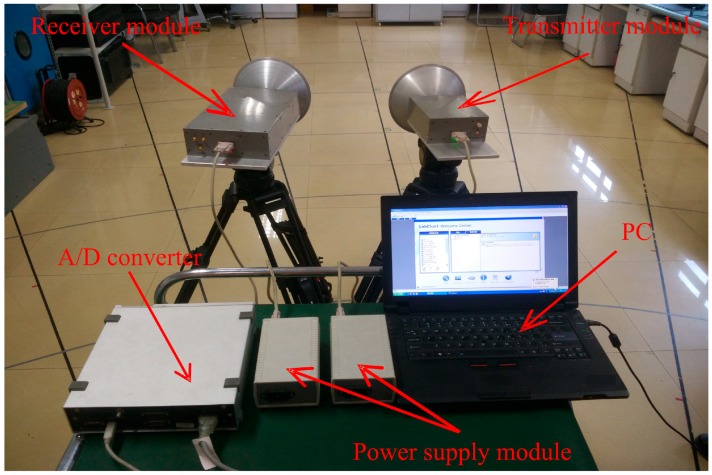
Photograph of the 94-GHz radar measurement system.

A photograph of the 94-GHz millimeter-wave radar detection system is shown in Figure 1. The system consists of a radar sensor, a personal computer (PC) controller, a 16-channel analogue-to-digital (A/D) converter, and two power supply units. The radar sensor includes a receiving antenna (Rx_Antenna), transmitting antenna (Tx_antenna), transmitter, and receiver module. The receiver module comprises a dielectric resonator oscillator (DRO), power amplifier with divider, frequency multipliers (×12), an isolator, low-noise amplifier (LNA), bandpass filter (86.7 GHz), and two downconverters (balanced mixer and I/Q mixer). The transmitter module contains frequency multipliers (×13), an isolator, a bandpass filter (94 GHz), an injection-locked amplifier (ILA), and a voltage-controlled variable attenuator. The transmitter module has dimensions of 205 × 145 × 70 mm and weighs 1.9 kg; the receiver module has dimensions of 360 × 185 × 70 mm and weighs of 4.1 kg.

The receiver and transmitter module are separate; they are each installed on a tripod equipped with a rotating head to enable alignment in any direction (Figure 1). The structure of the two separate antennas provides a high directivity gain, which can effectively increase the detection range and reduce the interference between antennas. Further, the structure enables the distance and angle between the two antennas to be easily adjusted, when directed to a target. Both the transmitting and receiving antennas are of the Cassegrain type with diameters of 200 mm each (ECA-W-10-200, ELVA-1 Company, St. Petersburg, Russia). The beam width is 1° × 1° at the –3 dB level in both horizontal and vertical directions. The operating wavelength is 3.19 mm. The waveguide band is WR-10, which is within the millimeter-wave atmospheric transmission window. Therefore, it can provide low propagation losses over long distances, when monitoring subtle physiological movements [22].

A continuous-wave signal is generated through a DRO at 7.23 GHz with an input power of 20 mW. This input local frequency (LF) signal is amplified and divided into the transmitting and receiving modules. In the transmitting module, the LF signal is multiplied and fed through an isolator, followed by a bandpass filter and an ILA, before being radiated by the transmitting antenna. For the receiver module, the LF signal is multiplied 12 times by the frequency multiplier. It is then fed through an isolator followed by a bandpass filter of 86.7 GHz. Owing to Doppler effect, the phase of the radar echo signal is modulated by the vibration of the human larynx. The echo signal is then balance-mixed with the processed signal at a frequency of 86.7 GHz. Next, the processed signal is amplified by an LNA, which is used to decrease the receiver noise figure by increasing the signal power at the input. The signal is then mixed with an LF signal to down convert it to the baseband signal in the I/Q mixer. The final baseband signal is sampled by the A/D converter, before reaching the computer. The I/Q mixer can avoid the null-point problem that normally occurs in single-channel radars.

The receiver with a superheterodyne architecture employs a two-step indirect conversion. In the first step, a single balanced mixer converts the signal to an intermediate frequency (IF). In the second step, the I/Q mixer converts the IF signal into a baseband signal. This structure can effectively mitigate severe DC offset problems and the associated 1/f noise in the baseband. The total gain of the RF-IF is 65 dB, and the I/Q phase balance is ±1°. The output radio frequency (RF) is 94 GHz with a power of 100 mW and the antenna gain is 41.7 dB. The maximum acceptable density, *S*, to which the human is exposed is approximately 0.3318 W/m^2^, when the distance between the 94-GHz radar and the subject is 1 m [18]. According to [23], the maximum allowed safe power density level is 10 W/m^2^ for human exposure at frequencies of 10–300 GHz. In addition, the 94-GHz frequency can penetrate skin to a depth of 0.37 mm which prevents penetration beyond the outer layer of the skin. That is to say, the electromagnetic wave power is predominantly reflected off the skin. Therefore, the electromagnetic radiation from the sensor does not pose risk to human health.

## 3. Radar Vibration Detection Theory for Vocal Folds

In a continuous-wave radar system, the transmitter sends a single-tone signal:

(1) $$P_{T}(t)=A\cos(2\pi f_{0}t+\phi (t))$$

where *f*_0_ is the carrier frequency, *φ*(*t*) is the oscillator phase noise, and *A* is the oscillation amplitude. Suppose the signal is directed at a subject’s larynx at nominal distance, *d*_0_, with a time-varying displacement, *x*(*t*). Then, the signal is phase-modulated by the motion of the larynx and is partly reflected it. The echo wave reaches the receiving antenna with a time difference that depends on the nominal distance and is detected by a sensitive receiver. The received signal is expressed as [24],

(2) $$P_{R}(t)=KA\cos(2\pi f_{0}t-\frac{4\pi d_{0}}{\lambda_{0}}-\frac{4\pi x(t)}{\lambda_{0}}+\phi (t-\frac{2d_{0}}{c}))$$

where *φ*(*t* – 2*d*_0_/*c*) is the received phase noise. *λ*_0_ = *c*/*f*_0_, and *K* is the decay factor of the oscillation amplitude. Then, the received signal and local oscillator signal are mixed:

(3) $$P_{M}(t)=A\cos(2\pi f_{0}t+\phi (t))\cdot [KA\cos(2\pi f_{0}t-\frac{4\pi d_{0}}{\lambda_{0}}-\frac{4\pi x(t)}{\lambda_{0}}+\phi (t-\frac{2d_{0}}{c}))]$$

The mixed signal is filtered by low-pass filtering. Thus, the signal can be expressed as,

(4) $$B(t)=\frac{KA^{2}}{2}\cos(\frac{4\pi d_{0}}{\lambda_{0}}+\frac{4\pi x(t)}{\lambda_{0}}+\Delta \phi (t))=\frac{KA^{2}}{2}\cos(\frac{4\pi x(t)}{\lambda_{0}}+\theta (t))$$

where $\Delta \phi (t)=\phi (t)-\phi (t-\frac{2d_{0}}{c})$ is the residual phase noise. *θ*(*t*) = 4*πd*_0_/*λ*_0_ + Δ*φ*(*t*).

Owing to the tiny glottis motion with an amplitude of several mm, the wavelength, *λ*_0_, of the 94-GHz radar is 3.19 mm. Hence, *x*(*t*) << *λ*_0_ is invalid; in this case, the small-angle approximation is invalid.

Using the Fourier series, any time-varying periodic displacement, *x*(*t*), can be viewed as the combination of a series of single-tone vibrations. The vibration of the vocal tract can be regarded as a quasi-periodic signal, which can be described by *x*(*t*) = *V*sin*wt*. Then:

(5) B(t)=Re{ej(4πVλsinwt+θ(t))}=Re{ej4πVλsinwt⋅ejθ(t)}=Re{[cos(4πVλsinwt)+jsin(4πVλsinwt)]⋅[cosθ(t)+jsinθ(t)]}=Re{cos(4πVλsinwt)⋅cosθ(t)−sin(4πVλsinwt)⋅sinθ(t)+jsin(4πVλsinwt)⋅cosθ(t)+jsinθ(t)⋅cos(4πVλsinwt)}=J0(4πVλ)cosθ(t)+2∑k=1∞J2k(4πVλ)cosθ(t)cos(2kwt)−2∑k=1∞J2k−1(4πVλ)sinθ(t)sin[(2k−1)wt)]

where *θ*(*t*) is the total residue phase. *J_k_*(*x*) is the *k*th-order Bessel function of the first kind. Thus, the phase-modulated baseband signal is decomposed into several frequency harmonics.

Note that the first part of the formula can be neglected because it represents a DC term, which does not involve successful detection. From Equation (5), when *θ*(*t*) denotes the odd multiples of *π*/2, the desired frequency is maximally recovered. When *θ*(*t*) denotes even multiples of *π*/2, the desired frequency vanishes. This situation is called a null-point problem, which commonly exists in Doppler radars. The null detection point occurs in every quarter wavelength from the radar to the subject. In the 94-GHz radar system, an I/Q quadrature receiver is used to alleviate this problem. Then, the output of the radar quadrature mixer can be expressed as [25]:

(6) $$B_{I}(t)=A_{I}\cos\left(\frac{4\pi x(t)}{\lambda_{0}}+\theta (t)\right)$$

and:

(7) $$B_{Q}(t)=A_{Q}\sin\left(\frac{4\pi x(t)}{\lambda_{0}}+\theta (t)\right)$$

If *A_I_* = *A_Q_*, the associated phase, *w*(*t*), can be extracted by:

(8) $$w(t)=\arctan\frac{B_{Q}(t)}{B_{I}(t)}=\frac{4\pi x(t)}{\lambda_{0}}+\theta (t)$$

Then, the displacement, *x*(*t*), and the amplitude information of the larynx can be extracted by processing the radar echo signal [17]:

(9) $$x(t)=\frac{\lambda_{0}}{4\pi }(w(t)-\theta (t))$$

(10) $$A_{R}(t)=\sqrt{{A_{I}}^{2}(t)+{A_{Q}}^{2}(t)}$$

## 4. Experimental

### 4.1. Experiment Set up

**Figure 2:**
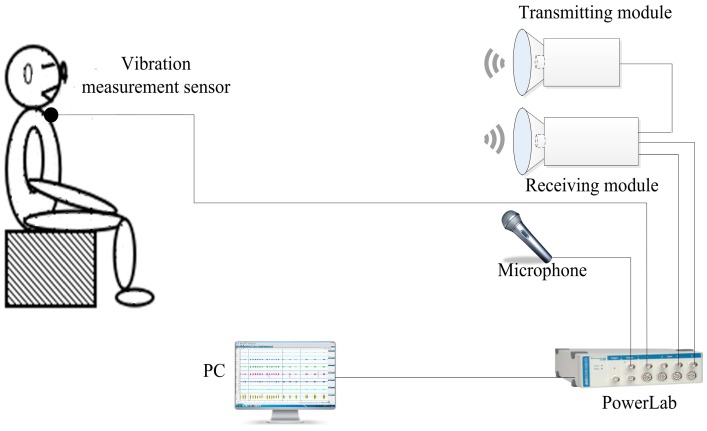
Schematic of the experimental system for detecting the vocal vibration signal.

The 94-GHz radar system, a vibration measurement sensor, and a microphone were used in this study to detect vocal vibration signals synchronously, as shown in Figure 2. A 16-channel data-acquisition PowerLab system (ADInstruments Pty. Ltd., Sydney, Australia) was connected to the control PC with a standard USB 2.0 interface. The PowerLab system was engineered for sampling the baseband signal with a full 16-bit ADC resolution. LabChart software (ADInstruments) was installed on the control PC to configure the acquisition parameters, acquire the data, and view the initial results.

All the signals were sampled at a frequency of 16 kHz and saved as a WAV file. The saved signals were then processed and analyzed using a MATLAB program (R2011a). A 12-VDC power supply provided power to the radar system. In the experiment, a volunteer was situated in front of the radar sensor. His throat was maintained at the same height as that of the radar system. The microphone system, used for the noncontact method and the vibration measurement sensor, used for the contact method was additionally incorporated to receive the vocal vibration signal for verification. The radar and microphone systems were positioned at distances ranging from 1 to 10 m from the subject. The vibration measurement sensor was adhered to the skin over the subject’s larynx. A laser pen was used to focus an electromagnetic beam on the larynx area.

Eight healthy volunteers including six males and two females, all native Chinese speakers, participated in the experiments. Their ages varied from 22 to 32, with a mean age of 27 and standard deviation of 9.17. None of them had a history of voice training or voice disorders. A variety of English vowels, words, and five sentences of Mandarin Chinese were used as the phonate material for acoustic analysis. Each participant spoke the sentences in a quiet experimental environment. All the experimental procedures were approved by the Ethical Committee of the Fourth Military Medical University in accordance with the rules of the Declaration of Helsinki [26]. All the participants provided written informed consent before the experiment.

### 4.2. Coherence Analysis of the Vocal Vibration Signal

Voice is composed of a fundamental frequency (pitch frequency) and harmonic frequencies resulting from the modulation of the vocal tract, tongue, lips, and jaw. To verify the advantage of the radar method in detecting the vibration signal from the vocal folds, coherence analysis was used to evaluate the correlation among the radar, vibration measurement sensor, and the microphone-detected signal in the frequency domain.

Coherence analysis can show the correlation of the voice signal in the pitch and harmonic frequencies. If *Rxx*(*τ*) and *Ryy*(*τ*) are the ACFs of *x*(*τ*) and *y*(*τ*), respectively, then the respective FFTs are *Pxx*(*f*), *Pyy*(*f*), and *Pxy*(*f*). The coherence function, *γ_xy_^2^*(*f*), is used for the conventional condenser microphone speech signal, *x*, and the new radar sensor speech signal, *y*, is defined as [27]:

(11) $${\gamma_{xy}}^{2}=\frac{{\left|P_{xy}(f)\right|}^{2}}{P_{xx}(f)P_{yy}(f)}$$

where the value of *γ_xy_^2^*(*f*) is between 0 and 1. The larger the value, the stronger is the relationship between the two input signals in the frequency domain.

## 5. Methods

The oscillation signal (pitch frequency) of the vocal folds is controlled by laryngeal mechanics and aerodynamic properties [28]. The vibration frequency of the vocal folds is one of the most important parameters in the voice signal; moreover, it describes an important feature of the speech excitation source. Therefore, the extraction of the vibration frequency of the vocal folds from the radar-detected signal is an important approach to evaluate the radar performance in the detection of the human vocal fold vibration. Further, a good estimation of the vibration frequency of the vocal folds is crucial for improving the performance of speech analysis and synthesis systems. The pitch frequency range of a normal voice signal is between 50 and 500 Hz [29].

**Figure 3:**
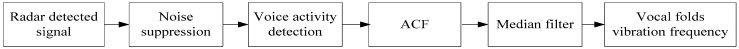
Block diagram of the signal-processing method.

After acquiring the radar-detected vocal vibration signal, a series of signal-processing methods were employed to detect the pitch period of the vocal vibration signal, as shown in Figure 3. First, EMD was employed to suppress the noise of the radar-detected signal explained in Section 5.1. Next, the ratio of the energy and entropy was employed to detect the voice activity, as detailed in Section 5.2. Third, a short-time ACF was employed to detect the vibration signal of the human vocal folds, as explained in Section 5.3. Finally, a median filter was used to minimize the effect of the outliers, as detailed in Section 5.4.

### 5.1. Noise Suppression by Empirical Mode Decomposition Algorithm

EMD is an adaptive method for processing nonlinear and nonstationary signals. In the process of decomposition, all the basic functions are derived from the signal itself. Therefore, the method is very well suited for processing nonlinear and nonstationary signals. For a radar-detected vocal vibration signal, if the noisy signal is represented by *y*(*n*), the noise-suppression process has the following steps:

1. Decompose the noisy signal, *y*(*t*), into intrinsic mode functions (IMFs) using the sifting process [30]. Then, the noisy signal can be expressed as:
  (12) $$y(t)=\sum_{i=1}^{n}IMF_{i}(t)+r_{n}(t)$$
  where *r_n_*(*t*) is the residual sequence.
2. Compute the mutual information entropy of the adjacent IMF components using the following equation:
  (13) MI(X;Y)=H(X)−H(X|Y)
  where *H*(*X*) represents the entropy.
3. For the radar-detected signal, the mutual information entropy of the adjacent IMF components is in the order of large to small and then to large. Hence, we can determine the cut-off point of the high- and middle-frequency modes.
4. Denoise the high- and middle-frequency modes with a soft thresholding function [31] as follows:
  (14) $$IMF_{i}^{\prime }(t)=\left\{ \begin{array}{ll} sign(IMF_{i}(t)-Thr_{i}) & \left|IMF_{i}(t)\right|\geq Thr_{i}\\ 0 & \left|IMF_{i}(t)\right|\leq Thr_{i} \end{array}\right.$$
  Here, *Thr_i_* denotes the adaptive threshold, which is estimated as:
  (15) $$Thr_{i}=\sigma_{i}\sqrt{2\log(N)}$$
  where *N* is the signal length and σ is the estimated noise level.
5. Reconstruct the IMFs with the noise-suppression signal and the remaining low-frequency modes with:
  (16) $$y(t)=\sum_{i=1}^{k}IMF_{i}^{\prime }(t)+\sum_{k+1}^{n}IMF_{i}(t)$$
  where *k* is the number of high- and middle-frequency modes, and *n* is the number of IMFs (see [18] for details on this algorithm).

### 5.2. Voice Activity Detection

If we segment a radar-detected speech signal represented by *x*(*n*) into *m* short-time frames, *x_i_*(*l*), each frame has a length of 320 samples with a 25% overlap [32]. Then, the *i*-th frame can be represented as a frequency by *X_i_*(*k*). The short-time frame can be regarded as a stationary sequence for further processing. The short-time energy of the *i*-th frame is defined as:

(17) $$E_{i}=\sum_{k=0}^{N/2}X_{i}(k)*X_{i}^{*}(k)$$

where *N* is the length of the fast Fourier transform and *k* denotes the *k*-th spectral line. The probability density function of the energy spectra can be described as:

(18) $$p_{i}(k)=\frac{X_{i}(k)*X_{i}^{*}(k)}{\sum_{k=0}^{N/2}X_{i}(k)*X_{i}^{*}(k)}\;k=0,1,\cdots ,N-1$$

From this point, the short entropy spectra of each speech frame are expressed as:

(19) $$H_{i}=-\sum_{k=0}^{N/2}p_{i}(k)\log p_{i}(k)$$

The ratio of the energy and entropy spectra, *R_i_*, is used as the voice activity function, described as:

(20) $$R_{i}=\sqrt{1+\left|E_{i}/H_{i}\right|}$$

In addition, the voice activity detection threshold, *T*, which is used to distinguish between speech frames and nonspeech frames can be determined by experiments. When the *R_i_-*value is greater than the *T-*value, the frame is a speech frame, otherwise it is nonspeech frame. In this paper, experimental results demonstrate that when the threshold is set to *T* = 0.12, this method provides better voice activity detection results.

### 5.3. Auto-Correlation Function Method

Short-time autocorrelation is a common method for estimating the pitch frequency. The basic concept of this method is to find the distance of the two maxima of the short ACF; the distance is the pitch period. The fundamental frequency is the reciprocal of the pitch period. For the radar signal, *x_i_*(*l*), the ACF is generally defined [33] as:

(21) $$R_{i}(k)=\sum_{l=1}^{N-k}x_{i}(l)x_{i}(l+k)$$

If *x_i_*(*l*) is an exact periodic signal with a period, *P*, then, *R*(*k*) is periodic with the same period:

(22) $$R_{i}(k)=R_{i}(k+P)$$

### 5.4. Median Filter

After the acquisition of the vibration signal of the vocal folds by the ACF method, certain outliers always affect the accuracy of the vibration frequency. In this study, a median filter is used to minimize the effect of the outliers. For a signal, *x*(*n*), if the length of the sliding widow is *M*, we can obtain the value of *M* from the series, *x*(*n* − *L*),…, *x*(*n* − 1), *x*(*n*), x(*n* + 1), …, *x*(*n* + *L*). Then, the signal can be obtained using the median filter expressed as:

(23) $$y(n)=median\{x(n-L),\cdots ,x(n),\cdots ,x(n+L)\}\;n\in Z,\;L=\frac{M-1}{2}$$

*M* is typically equal to three or five [34]; herein, it is equal to five.

## 6. Results

This section demonstrates the advantages of the noncontact radar in the detection of human vocal vibration signals. For comparison, two speech signal acquisition systems, including a microphone and a vibration measurement sensor, were additionally evaluated. The subject was requested to phonate the given text in a quiet experimental environment. Time-domain waveforms, frequency-domain spectra, and spectrograms were produced to evaluate the amount of noise, frequency distribution, and the fundamental and harmonic frequencies of the detected signal. Two English-language sounds, the vowel /a/ and the word “welcome” were selected for the evaluation. To guarantee high quality signals, a distance of 2 m between the subject and measurement system was selected as the representative distance.

**Figure 4:**
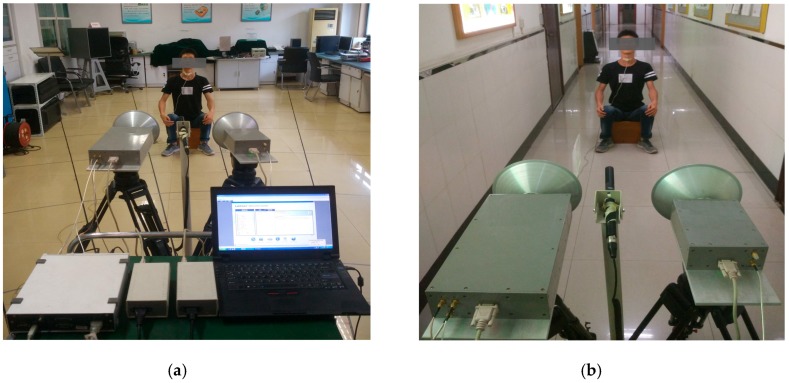
Experimental scenarios of the vocal vibration signal and acoustic signal detection: (**a**) enclosed room (2 m); (**b**) corridor (2 m).

The experiments were divided into two scenarios. The first scenario was in an enclosed room, as shown in Figure 4a. The second scenario was in a corridor. In these experimental scenarios, a 27-year-old male subject was located in front of the radar detection system. A microphone was placed on his throat at the same height as the radar antennas. Simultaneously, a vibration measurement sensor was adhered to the skin over the larynx. This sensor was used for acquiring the vocal vibration signal, particularly for detecting the vibration signal of the vocal folds.

**Figure 5:**
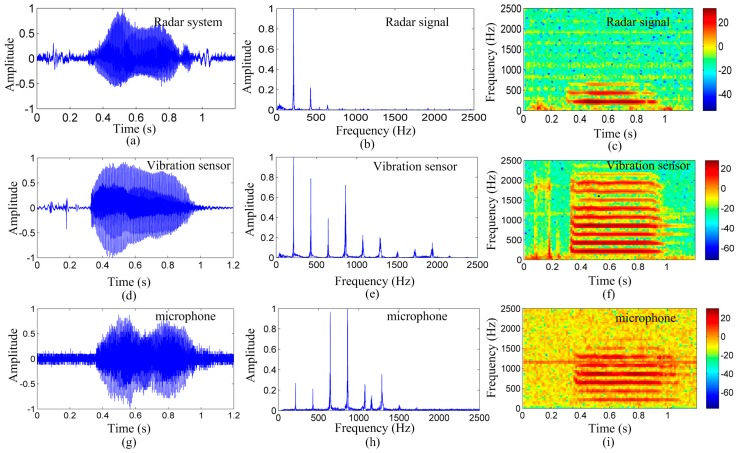
Measurement results of the radar system, vibration measurement sensor, and microphone system for the vowel /a/, as spoken by a young male subject. Time-domain waveforms, frequency-domain spectrum, and spectrograms of the (**a**–**c**): signal detected by the radar system, respectively; (**d**–**f**): signals detected by the vibration sensor, respectively; and (**g**–**i**): signal detected by the microphone system, respectively. From the spectrogram, the signal strengths of the different spectra over time are apparent. The color depth shows the signal energy value; the darker the color, the stronger is the signal energy.

Figure 5 shows the detection results of the radar system, microphone system, and vibration measurement sensor for the English vowel /a/, as spoken by the subject. Figure 5a–c depict the time-domain waveform, frequency-domain spectrum, and spectrogram of the radar system detected signal, respectively. Figure 5d–f show the time-domain waveform, frequency-domain spectrum, and spectrogram of the vibration measurement sensor detected signal, respectively. Figure 5g–i depict the time-domain waveform, frequency-domain spectrum, and spectrogram of the microphone-system detected signal, respectively.

As shown in Figure 5a,d,g, the time-domain waveform of the three measurement methods is considerably similar. This indicates that the proposed noncontact radar system effectively acquired the vocal vibration signal. As shown in Figure 5b,e,h, the normalized magnitude spectra of the detected signal of the three measurement methods are consistent for the first three peaks. The peaks represent the spectral line of the vocal vibration signal. The first peak represents the pitch frequency of the vocal-fold vibration; the normalized amplitude of the pitch frequency is the largest, for the signal detected by the radar and vibration measurement sensor, in particular. This indicates that the radar system and vibration measurement sensor may have effectively acquired the vibration signal of the vocal folds.

For the microphone system, the amplitude of the first peak is less than the amplitude of harmonics, indicating that the microphone system may have lost the vocal-fold vibration signal. Detailed results are shown in Figure 5c,f,i. The color of the lowest frequency components is lighter for the signal detected by the microphone system. Thus, when detecting the pitch frequency, the harmonics may be regarded as the vibration frequency of the vocal folds that limit the method’s accuracy in acquiring the vibration signal of the vocal cords. However, for signals detected by the radar system and the vibration measurement sensor, the color of the lowest frequency components is the darkest.

It is evident from Figure 5c,f,i that the three measurement methods are affected by some noise. Most of the noise signals exist in the time-domain waveform and spectrogram, particularly, for the microphone signal. This suggests that the 2-m testing distance was too far for the microphone. The vibration measurement sensor was placed directly on the skin over the larynx. Therefore, the noise in the vibration measurement sensor was less than that in the microphone. However, this can be easily disturbed by external factors. As shown in Figure 5f, certain unwanted signals exist at the starting. In contrast, the noise in the radar signal is less than that in the microphone signal, suggesting that the radar is essentially immune to acoustical disturbances at long detection distances. This characteristic makes it more suitable for applications in environments with high background acoustic noise, where the acoustic signals are inaccessible or blocked. These results suggest that the radar system is superior in detecting vocal vibration signals over long distances compared to the microphone system and the vocal measurement vibration.

**Figure 6:**
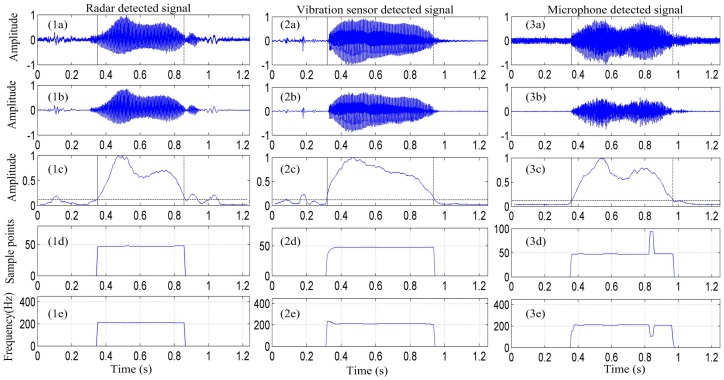
Pitch contours of the signals detected by the radar system, vibration sensor, and microphone system for the vowel sound /a/ obtained using the EMD-ACF algorithm. (**a**) Original detected signal; (**b**) enhanced speech obtained using the EMD method; (**c**) voice activity detection using the ratio of the energy and entropy spectra; (**d**) pitch period of the detected signal; and (**e**) vocal-fold vibration frequency of the detected signal.

To verify the performance of the radar detection system in detecting the vibration signal from vocal folds, the proposed algorithm combining the EMD and ACF was used to extract the vocal cord vibration frequency from the detected signal. Figure 6 depicts the vocal-fold-vibration frequency contour of the signals detected by the radar, vibration measurement sensor, and microphone for the sound of the English vowel /a/ obtained using the EMD-ACF algorithm.

Figure 6(1a–3a) show the signal of the /a/ sound is detected by the radar, vibration sensor, and microphone, respectively. Figure 6(1b–3b) depict that the signal is enhanced by the EMD algorithm. Figure 6(1c–3c) present the signal-activity detection result. Figure 6(1d–3d) show the pitch-period contour of the detected signal obtained using the EMD-ACF algorithm, and Figure 6(1e–3e) depict the corresponding vocal-fold vibration frequency.

Further, in Figure 6, it is apparent that the proposed EMD-ACF algorithm for detecting the vibration frequency of vocal folds performs well at different signal-to-noise ratios. In Figure 6b, the noise in the three measurement methods of the detected signal is effectively removed by the EMD algorithm. The average vocal-fold vibration frequency of the signal detected by the radar is approximately 212 Hz, and the average vocal-fold vibration frequencies detected by the vibration sensor and microphone are approximately 212 Hz and 203 Hz, respectively. This suggests that the vocal-fold vibration frequency in the radar-detected signal is consistent with that in the signals detected by the microphone and vibration measurement sensor. However, some outliers exist in the vocal-fold vibration contour of the microphone system, which may be due to the signal containing certain some low-frequency disturbance signals, a major source of pitch error.

The superior performance of the radar system and vibration measurement system is attributed to their methods for obtaining the vocal-fold vibration signal. Moreover, the former system is superior to the latter because it can achieve noncontact detection over a long distance. This is because the radar system can directly detect small displacements for the effective direction sense of the millimeter wave. This capability provides the radar system with high anti-jamming abilities in noisy environments.

**Figure 7:**
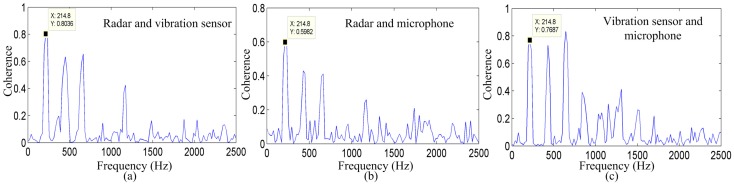
Coherence among the three measurement methods for the vowel sound /a/. Coherence between the signals detected by the (**a**) radar and vibration sensor; (**b**) radar and microphone system, and (**c**) vibration sensor and microphone system.

Figure 7 shows the coherence between the radar system and microphone system, the radar system and vibration measurement sensor, and between the microphone system and vibration measurement sensor for the vowel sound /a/. Significant coherence is evident in the vocal-fold vibration frequency and in some harmonics, among the three measurement methods that exhibit a peak in the spectra at approximately 214.8 Hz. This peak must be the vocal-fold vibration frequency.

As shown in Figure 7a–c, the coherence values between the radar system and vibration measurement sensor, the radar system and microphone system, and between the vibration measurement sensor and microphone system are 0.8036, 0.5962, and 0.7687, respectively. In addition, Figure 7 shows the coherence of the same vowel sound, indicating that the energy distribution of the signal detected by the radar corresponds well to the energy distribution of the signal detected by the vibration sensor at most frequencies. These results suggest that there is a clear similarity in the vocal-fold vibration frequency and harmonics among the three measurement methods.

**Figure 8:**
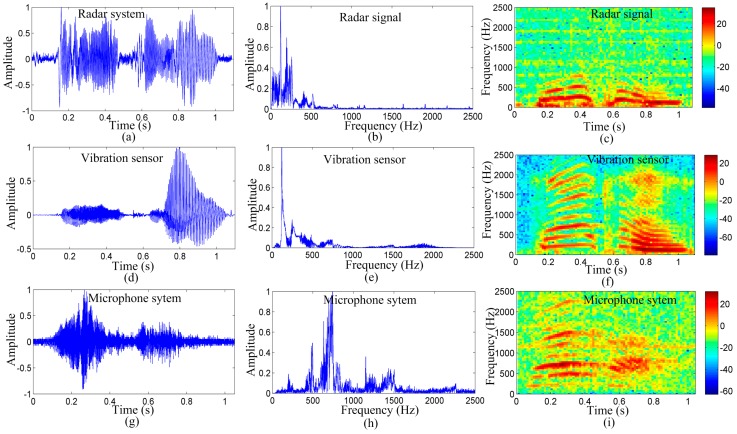
Measurement results of the radar system, vibration measurement sensor, and microphone system for the word “welcome” as spoken by a young adult male. Time-domain waveforms, frequency-domain spectrum, and spectrograms of the signal detected by, (**a**–**c**) the radar system, respectively; (**d**–**f**) vibration sensor, respectively; and (**g**–**i**) microphone system, respectively.

An additional experiment was conducted for word detection. Figure 8 shows the detection results of the radar system, microphone system, and vibration measurement sensor for the word “welcome”. From the figure, it is evident that there are certain differences in the time-domain waveforms among the signals detected using the three measurement methods. However, in the spectrograms, the radar system, vibration sensor, and microphone are consistent in their distribution patterns.

As shown in Figure 8c,f,i, we can determine that the noise of the signal detected by the radar is less than that of the signal detected by the microphone. The low-frequency component, particularly, the fundamental frequency, is lost because of the long distance. However, as demonstrated by the frequency-domain information, the energy of the vocal-fold vibration frequency signal is very high for the radar and vibration sensor, particularly, for the signal detected by the radar. Figure 8b,e,h show that the vocal-fold vibration frequency of the signal detected by the radar and vibration sensor are the same, and are different from that of the signal detected by the microphone.

**Figure 9:**
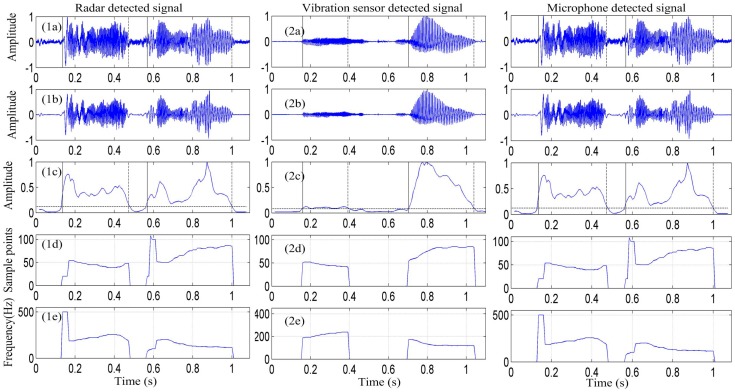
Pitch contours of the signals detected by the radar system, vibration sensor, and microphone system for the spoken word, “welcome,” obtained using the EMD-ACF algorithm. (**a**) Original detected signal; (**b**) enhanced speech obtained using the EMD method; (**c**) voice activity detection using the ratio of the energy and entropy spectra; (**d**) pitch period of the detected signal; and (**e**) vocal-fold vibration frequency of the detected signal.

Figure 9 shows the pitch contours of the signals detected by the radar system, vibration sensor, and microphone system for the spoken word, “welcome,” obtained using the EMD-ACF algorithm. The results obtained from this figure are the same as those obtained from the previous experiment. The pitch period and vocal-fold vibration frequency contour of the signal detected by the radar are similar to those of the signals detected by the microphone and vibration measurement sensor.

**Figure 10:**
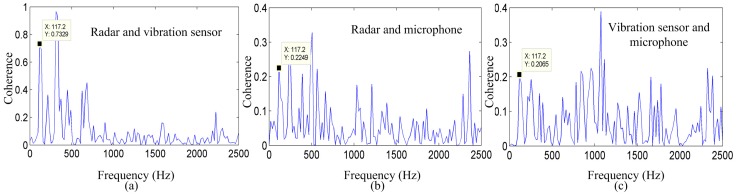
Coherence between the three measurement methods for the spoken word “welcome.” Coherence between the signals detected by the (**a**) radar and vibration sensor; (**b**) radar and microphone system; and (**c**) vibration sensor and microphone system.

Figure 10 presents the coherence between the radar and microphone system, the radar system and vibration measurement sensor, and between the microphone system and vibration measurement sensor for the spoken word, “welcome.” A high coherence is apparent in both the vocal-fold vibration frequency and harmonics between the signals detected by the radar and vibration sensor. However, the coherence is low between the signals detected by the radar and the microphone, and between the signals detected by the vibration sensor and microphone. These results indicate that the radar system is more appropriate for detecting vocal vibration signals over long distances, particularly for acquiring detailed information on the vocal-fold vibration signal.

To further demonstrate the effectiveness of the radar system in the detection of the vibration signal from human vocal folds for long detection distances, Table 1 shows the detection results of the radar system, microphone system, and vibration measurement sensor for the English vowel /a/, as spoken by the subject when the distance between the radar system and the subject is 1, 2, 5 and 7 m, respectively. From the table, it can be observed that the coherence values show a high consistency in the vocal-fold vibration frequency among the three measurement methods, when the distance between the radar system and the subject is 1, 2, 5 and 7 m. It also can be seen from the table that when the distance is 7 m, the coherence between the radar system and the vibration sensor is still high; however, the coherence value is relatively low between the radar system and microphone, and between the microphone and vibration sensor. This is because the microphone may have lost certain detailed information, which is the vocal vibration frequency.

In order to test the effectiveness of the radar system in detecting different words, a variety of English alphabet letters “A”, “B”, “C”, ”D” and one sentence of Mandarin Chinese, “Jun-Yi-Da-Xue”, spoken by a 22-year-old male subject were also tested for verifying the reliability of the aforementioned results. The distance between the radar system and the subject was 2 m. The coherence results between the radar and microphone system, radar system and vibration measurement sensor, and between the microphone system and vibration measurement sensor for these spoken words are presented in Table 2. It can be seen from the table that significant coherence is still evident in the vocal-fold vibration frequency and certain harmonics among the three measurement methods, which further verifies the effectiveness of the radar system in detecting the vibration signal from human vocal folds.

## 7. Discussion

In this article, a noncontact 94-GHz millimeter-wave radar system was proposed for measuring the human vocal vibration signal. By comparing the vocal vibration signals synchronously measured with the microphone and vibration measurement sensor, we determined that the signal detected by the radar is consistent with the signals detected by the microphone system and vibration measurement sensor in the time domain, frequency domain, and spectrograms. This was particularly the case for the vocal-fold vibration frequency and the second and third harmonics signals, while the higher-frequency signals were diminished.

As shown in Figure 5c,f,i, and in Figure 8c,f,i, the energy distribution of the signal detected by the radar in the vocal-fold vibration frequency was stronger than those of the signals detected by the microphone and vibration measurement sensor. The signal detected by the microphone may have losses in the vocal-fold vibration frequency. Furthermore, Figure 5 and Figure 8 exhibit less noise in the signal detected by the 94-GHz radar for both the time waveform and spectrograms.

These results demonstrate that the proposed 94-GHz radar system has an advantage over the microphone system and vibration senor in the measurement of the human vocal vibration signal, particularly in the detection of the vocal-fold vibration over a long distance. This is because the 94-GHz millimeter-wave radar has a beam width of 1° × 1° at the –3 dB levels in both the horizontal and vertical directions. This feature provides the system the advantage of good directional and anti-jamming capabilities. In addition, the high operating frequency provides greater sensitivity to small displacements, such as tiny vocal vibration displacements, which are typically in the order of mm. Thus, the signal detected by the radar is mainly modulated by the skin covering the vocal-fold area. However, the signals detected by the microphone and vibration measurement sensor may be modulated by human articulators such as the vocal folds, tongue, lips, and jaw. These results indicate that this radar system can directly detect the vocal vibration signal at a long detection distance.

An interesting experiment was additionally conducted for obtaining further evidence to substantiate our findings. Two volunteers were placed 2 m in front of the radar system; one of the volunteers was located directly in front of the radar system, which can be regarded as a 12-o’clock direction; whereas, considering the radar system as the center, with a 2 m radius, the other volunteer was located in the 1-, 2- and 3-o’clock directions, respectively. The larynx of the two volunteers were maintained at the same height as the radar system. The two volunteers spoke two different sentences. Meanwhile, the microphone system and vibration measurement sensor simultaneously detected the signal. The results showed that the radar system could effectively detect the desired vocal vibration signal; moreover, the detected signal was not disturbed by the other signals. However, the signals detected by the microphone and vibration sensor were totally disturbed by undesired signals.

In conclusion, in this paper, we have a proposed a noncontact method for detecting the vocal-fold vibration signal. The results have demonstrated the advantages of the radar system in long-distance detection, preventing acoustic disturbances, and ensuring high directivity. The energy of the signal detected by the radar was mainly distributed in the low-frequency ranges. This could be attributed to the effects of the 94-GHz operating frequency that improves the detection sensitivity of the radar sensor for small vibrations from the vocal folds. The proposed radar technology has promising applications in environments with high background acoustic noise and will likely be used to diagnose articulator disorders and calculate transfer functions for speech coding, speech recognition, and speaker verification.

## Figures and Tables

**Table 1:** Results of coherence between the radar and microphone, radar system and vibration sensor, and between the microphone and vibration sensor for the spoken English vowel /a/, when the distance between the radar system and the subject is 1 m, 2 m, 5 m and 7 m, respectively.

Distance (m)	1	2	5	7
**Pitch Frequency (Hz)**	214.8	214.8	214.8	214.8
**Coherence Value (Radar and Sensor)**	0.74	0.80	0.98	0.88
**Coherence Value (Radar and Microphone)**	0.93	0.61	0.62	0.47
**Coherence Value (Sensor and Microphone)**	0.71	0.77	0.56	0.56

**Table 2:** Coherence results between the radar and microphone, radar system and vibration sensor, and between the microphone and vibration sensor for the spoken English letters “A”, “B”, “C”, and ”D” and a sentence of Mandarin Chinese, “Jun-Yi-Da-Xue”.

Alphabet/Word	A	B	C	D	Jun	Yi	Da	Xue
**Pitch Frequency (Hz)**	253.9	285.5	302.7	166	283.2	263.7	195.5	195.3
**Coherence Value (Radar and Sensor)**	0.86	0.51	0.91	0.97	0.55	0.30	0.69	0.92
**Coherence Value (Radar and Microphone)**	0.26	0.59	0.45	0.61	0.44	0.26	0.31	0.69
**Coherence Value (Sensor and Microphone)**	0.26	0.71	0.36	0.55	0.48	0.63	0.93	0.63
